# Determination and Identification of Antibiotic Drugs and Bacterial Strains in Biological Samples

**DOI:** 10.3390/molecules25112556

**Published:** 2020-05-31

**Authors:** Katarzyna Pauter, Małgorzata Szultka-Młyńska, Bogusław Buszewski

**Affiliations:** 1 Department of Environmental Chemistry and Bioanalytics, Faculty of Chemistry, Nicolaus Copernicus University, Gagarin 7, 87-100 Torun, Poland; kpauter@wp.pl (K.P.); bbusz@umk.pl (B.B.); 2 Centre for Modern Interdisciplinary Technologies, Nicolaus Copernicus University, Wilenska 4, 87-100 Torun, Poland

**Keywords:** antibiotics, biological samples, analytical techniques, mass spectrometry, microorganisms

## Abstract

Antibiotics were initially natural substances. However, nowadays, they also include synthetic drugs, which show their activity against bacteria, killing or inhibiting their growth and division. Thanks to these properties, many antibiotics have quickly found practical application in the fight against infectious diseases such as tuberculosis, syphilis, gastrointestinal infections, pneumonia, bronchitis, meningitis and septicemia. Antibiotic resistance is currently a detrimental problem; therefore, in addition to the improvement of antibiotic therapy, attention should also be paid to active metabolites in the body, which may play an important role in exacerbating the existing problem. Taking into account the clinical, cognitive and diagnostic purposes of drug monitoring, it is important to select an appropriate analytical method that meets all the requirements. The detection and identification of the microorganism responsible for the infection is also an essential factor in the implementation of appropriate antibiotic therapy. In recent years, clinical microbiology laboratories have experienced revolutionary changes in the way microorganisms are identified. The MALDI-TOF MS technique may be interesting, especially in some areas where a quick analysis is required, as is the case with clinical microbiology. This method is not targeted, which means that no prior knowledge of the infectious agent is required, since identification is based on a database match.

## 1. Introduction

Microbiology is a leading science branch, which is particularly important for medicine, biotechnology, veterinary studies and agriculture. There is no doubt that microorganisms play an extremely significant role in the human life and surrounding environment. Moreover, an increasingly important role in the process of ontogenesis is attributed to the influence of various microorganisms: viruses, bacteria and fungi [1,2]. The detection of the infection in its early stages could help achieve better outcomes, and therefore, it is extremely important not only to determine changes within the body, but also to find biomarkers that characterize a given individual or population. Hence, in recent years, an increasing emphasis has been placed on the search for modern, very precise, and, above all, quick methods for the identification of microorganisms along with antibiotic drugs and their metabolites. This requires an interdisciplinary approach, in which the cooperation of specialists in medicine, chemistry, biochemistry, microbiology, molecular biology, and bioinformatics will allow the scientists to determine the immunological correlation between certain microorganisms and biomarkers (protein markers, volatile organic compounds) and the occurrence of diseases.

Over the last few years, the focus has been made on combined techniques as the main tools for solving complex analytical problems. Separation techniques (LC, GC, CE) coupled with different detection systems (FTIR, PDA, NMR and MS) permit the identification of compounds present in the raw sample. These techniques include LC-FTIR, LC-NMR, LC-MS, HPLC-PDA, HPLC-MS, GC-NMR and GC-FTIR [3]. What is especially remarkable, is that the studies carried out so far on different species and strains of microorganisms indicate a strong potential for the use of hyphenated separation techniques, especially CE-MS and GC-MS [4]. Therefore, a key step will consist in evaluating the usefulness of electromigration techniques and the technology of matrix-assisted laser desorption/ionization with time of flight (MALDI-TOF MS) for the identification and characterization of native microbial cells. In addition, the use of multi-dimensional, coupled separation techniques (LC × LC-MS/MS, GC × GC-Q-TOF/MS and LC × LC-CZE-MS/MS) will offer the possibility to prepare the metabolomic profiling of the studied biological samples in order. This techniques can be of two types—on-line or off-line procedure integration. The off-line mode is an appropriate solution for the optimization of method parameters and does not require any additional technical equipment. Although the off-line mode is very flexible, it has disadvantages in time and labor consumption, the possibility of loss or contamination of the sample and a large sample volume that may not be suitable for a very sensitive identification of proteomic samples. The alternative is to use the on-line system, which automates the sample preparation process and thus, it reduces the time of the analysis [3,5]. Moreover, the application of statistical methods will facilitate a detailed and multi-directional interpretation of the data, which can significantly contribute to progress in the detection and treatment of diseases caused by pathogens.

## 2. Antibiotic Drugs

Antibiotics always existed in our environment, but we did not know how to isolate and produce them. The first potentially healthy use of beer containing tetracycline was found in ancient Nubia about 350–550 years BC [6]. The modern era of antibiotics began with Alexander Fleming (1881–1955), the great antibiotic explorer. The most famous phrase of Fleming is: “Penicillin was produced by nature, I only discovered it” [7]. Today, it is estimated that there are more than 70,000 natural antibiotics [8].

Antibiotics were initially natural but nowadays they also have synthetic substances showing activity against bacteria, killing or inhibiting their growth and division. Thanks to these properties, many antibiotics have quickly found practical application in the fight against infectious diseases such as tuberculosis, syphilis, gastrointestinal infections, pneumonia, bronchitis, meningitis and septicemia [9].

**Figure 1:**
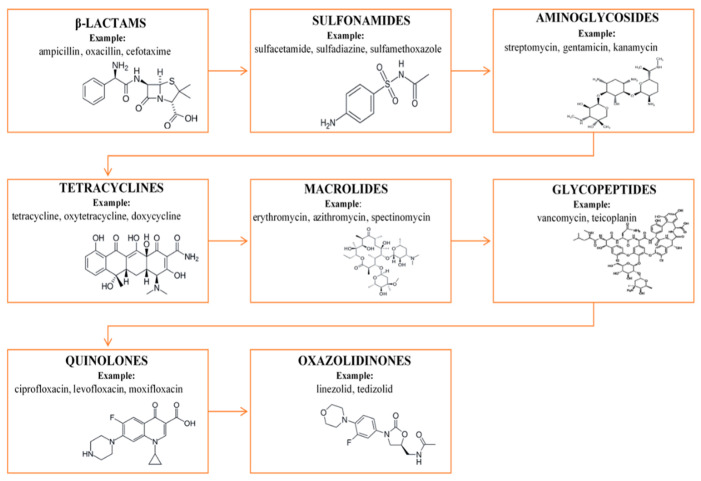
An overview of main antibiotics.

From a chemical point of view, antibiotics form different groups of compounds. In general, they are low molecular weight compounds, characterized by different chemical structures, composition and physicochemical properties. A well-known group of antibiotics are β-lactams which include penicillins and cephalosporins. Other classes of antibiotics consist of macrolides, amidoglycosides, sulfonamides and tetracyclines [10]. In Figure 1, the main classification of antibiotics is presented. Moreover, antibiotics can be divided into classes with broad or narrow antibacterial spectra. Most of the studied antibiotics are active against Gram-positive bacteria and a smaller number against Gram-negative bacteria [10]. Analyzing the effect of antibiotics on bacteria, there are bactericidal (vancomycin, amoxicillin, cefuroxime) and bacteriostatic (cycloserine, linezolid, azithromycin) antibiotics. Bacteriostatic drugs cause a reversible inhibition of growth, with bacterial culture restarting after the elimination of the drug. By contrast, bactericidal drugs kill their target bacteria.

The strong antimicrobial activity of antibiotics led to the study of the mechanisms of this phenomenon. Some mechanisms of their action proved to be varied, and their place of activity may be a cell wall, whose synthesis at various stages of its formation is disturbed by penicillins, cephalosporins, cycloserine, vancomycin, and other antibiotics. The essence of the antibacterial action of these drugs is to inhibit the formation of bridges connecting the subunits of peptidoglycan into an integral whole. This process is catalyzed by bacterial enzymes called penicillin-binding proteins (PBP), located in the cell membrane of bacteria that bind the antibiotic. As a result of a permanent binding with an antibiotic, the function of enzymes (PBP) is blocked and, as a result, the cell maturation and the cell division are inhibited [11].

The other antimicrobial agents (polymyxins, polyesters) influence the breakdown of the cell membrane and the increase in its permeability to ions. Antibiotics acting on the cell membrane have a specific structure that allows them to bind with the lipid components of the cell membrane, causing the membrane to lose its tightness. Daptomycin is also an antibiotic disturbing the cell membrane functioning. The mechanism of the action of daptomycin is based on its irreversible binding with the cell membrane of Gram-positive bacteria, in the presence of calcium ions. The effect of this action is the formation of channels leading to the depolarization of the cell membrane and the outflow of potassium and other ions from the cell interior. As a result of this process, the membrane is destroyed and the macromolecular synthesis of macromolecules is seriously disturbed [12].

A relatively large group of various antibiotics inhibits protein synthesis at many stages, from the initiation of translation to the proper chain elongation. The protein synthesis is associated with special cellular structures called ribosomes. The bacterial ribosome consists of ribonucleic acids (rRNA) and proteins. It dissociates into two subunits—large (50S) and small (30S). Due to the many classes of antibiotics inhibiting protein synthesis, the molecular mechanisms of their action are different. Usually, drug molecules bind to different ribosomal protein molecules or ribosomal RNA molecules in both the 30S and 50S subunits, causing the cell death. The group of drugs disturbing protein synthesis includes aminoglycosides and macrolides [13].

The basic class of antibiotics disturbing the DNA synthesis are quinolones and their derivatives such as fluoroquinolones (II and III generation of drugs). These antibiotics are specific inhibitors of topoisomerase II ligase domains (gyrases) and IV topoisomerase domains. As a result of the nucleolytic domain’s activity, the DNA in the cell is fragmented [14]. In addition to the effect on the DNA synthesis and half-life, there is a group of antibiotics that affect the RNA synthesis (ansamycins), which includes the widely known rifampicin. It binds specifically to the bacterial RNA polymerase in the vicinity of the active site and prevents the RNA chain elongation. Antibiotics (sulfonamides) may also affect the activity of important metabolic pathways in the cell. One of the best-known examples is the inhibition of folic acid synthesis, which leads to a disruption of the DNA synthesis [15].

## 3. Drug Metabolism 

**Figure 2:**
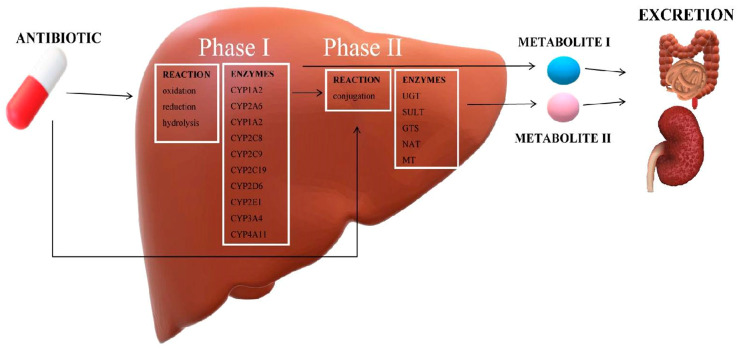
The concept of antibiotics’ metabolism in the liver.

Each active substance that we deliver to our body must undergo several stages in order to obtain the appropriate pharmacological effect. Antibiotics undergo the biochemical modification (biotransformation) not only in the liver, but also in the kidneys, blood and small intestine walls. The metabolism of antibiotics involves different processes, which are divided into phase I and phase II reactions (Figure 2) [16]. Phase I reactions lead to the formation of intermediates in the processes of oxidation, reduction and deamination. Phase II reactions, on the other hand, consist in coupling the end products with the glucuronic acid, the sulphuric acid, glutathione and glycine, and may lead to methylation or acetylation. Phase I takes place mainly by oxidoreductases and hydrolysis, as opposed to phase II, which takes place by means of transferases catalyzing coupling reactions (glucuronosyltransferase) or cytosolic enzymes (sulfotransferase, N-acetyltransferase). The metabolites formed in this way differ in their properties and can be divided into active, inactive, toxic or those that are transformed into a primary compound under the influence of appropriate physical factors. Depending on the type of the metabolite produced, the antimicrobial activity and toxicity of the primary compound may increase, which may result in certain restrictions in the use of antibiotics [17].

In most cases, under the influence of biotransformation, inactive compounds are formed, which in a short period of time are eliminated from the body. However, active metabolites may be formed from both active and inactive parent compounds. When a non-active drug is transformed into an active metabolite in the body, the conversion of prodrug to drug takes place. Where the parent is an active medicine, the resulting active metabolites may have synergistic or additive effects and may prolong and/or enhance its effects. These metabolites may have lower toxicity than the parent compound or a different pharmacological activity. This feature is visible in the development of new drugs. Metabolic processes may also result in the formation of toxic substances which increase the harmfulness of the medicine used, or in the formation of metabolites which reenter the parent compound under appropriate environmental conditions. Depending on the type of metabolite formed, the antimicrobial activity or the toxicity of the parent compound may increase, which may result in a number of restrictions related to taking the drug [17].

Penicillins are a widely used group of antibiotics. Isoxazolyl penicillins are described in more detail in all groups of penicillins. Their biotransformation results in para-hydroxy and 5-hydroxymethyl derivatives, which show a partial activity of the parent compound. However, the highest activity is observed in the oxacylin metabolite, which, isolated from urine, retains 10–20% of the activity of the primary compound. The antimicrobial activity of all metabolites originating from isoxazolyl penicillins is twice as low as that of their parent compounds. However, all substances remain active against *Staphylococcus* genus bacteria resistant to benzylpenicillins [18].

Metronidazole is a chemotherapeutic agent from the nitroimidazole group. Its biotransformation results in hydroxymethylmetronidazole. This metabolite has twice as high an activity as the parent compound in relation to bacteria of the genus *Gardnerella vaginalis* and a similar activity in relation to Gram-positive *Staphylococci* [19].

Among the antibiotics belonging to the group of lincosamides, clindamycin and its active metabolites are noteworthy. The transformation of the parent compound results in the formation of two main compounds that show an antimicrobial activity. These are clindamycin sulfoxide and *N*-demethylclindamycin. A metabolite with a higher antimicrobial activity is *N*-demethylclindamycin. This compound has twice as high an antimicrobial activity as clindamycin [20]. The antibacterial activity of selected antibiotic metabolites is presented in Table 1 [21,22,23,24,25,26,27,28].

Substances remaining in the tissues, and those which are released to the environment, can lead to the induction of bacterial resistance. Antibiotic resistance is currently a detrimental problem, therefore, in addition to the improvement of antibiotic therapy, attention should also be paid to active metabolites in the body, which may play an important role in exacerbating the existing problem.

## 4. Antibiotic Resistance

Antibiotics were considered a miracle cure for many years. The pioneers of antibiotic therapy believed that these antimicrobial agents would eliminate all bacterial infections. Unfortunately, bacteria struggling to survive revealed a wide range of possibilities to gain resistance to drugs. The first signal was a large group of penicillin resistant *Staphylococcus aureus* strains, and in the early 1960s, methicillin resistant strains (MRSA) were observed [9]. The resistance of microorganisms to the introduced class of antibiotics appeared almost immediately after their first use in therapy. Such a development was predicted by Alexander Fleming. During his work on penicillin, he noted that successive generations of *Staphylococcus aureus* treated with penicillin produced cell walls that are less and less permeable to this drug. Therefore, he discovered one of the mechanisms of antibiotic resistance [29].

Antibiotic resistance can be determined from the genetic information encoded in a chromosome or in moving elements such as plasmids, transposons and integrons. Bacteria may be naturally resistant to a specific group of antibiotics or may acquire resistance through various genetic processes, including mutations, resistance gene transfer, and also through direct contact with cells. The transfer of immune genes takes place through the horizontal gene transfer. Vectors carrying such genes are usually plasmids (called plasmids *R*-resistance), which in conjugative processes can be transferred from the donor cell to the recipient cell. Antibiotic resistance can also be determined by mobile genetic elements such as transposons or integrons, which are one of the sources of bacterial strains resistant to several chemotherapists at the same time. The variability of the genome leads to a change in cell metabolism, which results in the appearance of enzymes with a wide spectrum of action, including inactivating antibiotics. Another factor of the mechanism of antibiotic resistance is the fact that bacteria have elution pumps at their disposal. These pumps located in the cytoplasmic membrane are proteins transporting toxic substances, including antibiotics, outside the bacterial cell. Efflux pumps are present in both Gram-positive and Gram-negative bacterial cells. They are an important tool for initiating antibiotic resistance, including the development of multifactorial resistance [30].

According to Acar and Moulin [31], the ability of bacteria to acquire antibiotic resistance depends on the ability of individual bacteria to adapt to the selective pressure of the antibiotic used. In the classification proposed by them, the following mechanisms of drug resistance were distinguished:

1. The active efflux, which prevents the achievement of the antibiotic target, i.e., the place where the function of the bacterial cell is damaged;
2. The reduction of the permeability of the bacterial cell membrane, which occurs when its composition and function are modified;
3. The modification of an antibiotic in its inactive form with the participation of enzymes produced by bacteria; they may change the antibiotic inside or outside the bacterial cell, removing its antibacterial effect;
4. The change of the target of the antibiotic, reducing its affinity to it;
5. Bacterial mutations resulting in the elimination of bacteria resistant by the antibiotic;
6. The occurrence of a mixed population of sensitive and resistant bacteria at antibiotic concentrations on the selection of resistant cells [31].

**Figure 3:**
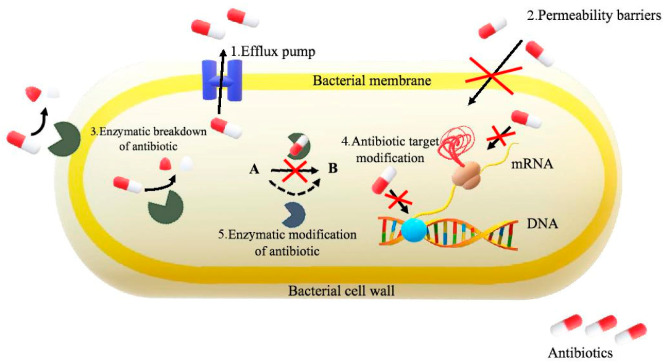
Molecular mechanism of antimicrobial resistance.

Molecular mechanisms of antimicrobial resistance in bacteria are provided in Figure 3. The reason for antibiotic resistance is, therefore, the evolution and exchange of genetic material through the so-called horizontal gene transfer and selection, which is, unfortunately, mainly caused by human activity. Human influence results in an improper intake of antibiotics and their use as a food additive for animals. Unfortunately, the development of antibiotic resistant strains of microorganisms is still an unresolved problem.

## 5. Determination and Identification of Antibiotic Drugs and Their Metabolites

### 5.1. Microbiological Assay

A rapid recognition of a life-threatening infection, as well as a reliable identification of the pathogen causing it and the selection of the most effective antibiotic are key factors to achieve a therapeutic success. The choice of an antimicrobial drug and its lowest concentration that prevents the visible growth of bacteria are based on the minimum inhibitory concentration (MIC). It is a value measured in vitro, which allows to determine the drug susceptibility of microorganisms. Disk diffusion, dilution methods, E-test and automated systems are commonly used MIC measurement techniques. There are two main types of dilutions: micro- and macro-dilution, with broth and agar being the most frequently used media. In the early 1870s, dilution was one of the earliest methods in the microbiological practice [32,33].

In the clinical practice, the quantitative determination of antibiotics is one of the more complex areas of the pharmaceutical analysis, especially in patients with infections difficult to treat, e.g., endocarditis. In this method, the same number of bacterial cells is added to the liquid or solid medium with the antibiotic at a certain concentration, and the growth in the presence of the antibiotic is assessed. The serial dilution method also makes it possible to determine the lowest bactericidal concentration (MBC). Currently, the main MIC determination method used in routine testing in medical microbiology laboratories around the world is the gradient diffusion method Epsilometer test (E-test^®^). It combines the principle of agar antibiotic diffusion with the determination of the minimum inhibitory concentration of the antibiotic by dilution in agar. Quantitative testing with the E-test is based on the diffusion of the antibiotic from the tissue paper strip, in the concentration gradient, to the medium on which the bacteria strain grows. The antibiotic gradient strip diffusion method is applicable to both the MIC determination for fast-growing aerobic bacteria such as Staphylococci, Gram-negative Enterobacteriaceae and demanding bacteria such as *Streptococcus pneumoniae* and anaerobic bacteria [34,35].

Kontopidou et al. [35] studied the antibiotic susceptibility of bacterial isolates from bronchial secretion samples. The E-test and disk diffusion were compared with the dilution technique to determine in vitro activity of five antibiotics (ciprofloxacin, piperacillin, tazobactam, meropenem and colistin). Both direct diffusion tests (E-test and disk diffusion) were susceptible to interception and could be helpful in improving the treatment of Ventilator-Associated Pneumonia (VAP) [35].

Di Bonaventura et al. [34] used E-test^®^, agar/broth dilution and disk diffusion methods for testing the levofloxacin susceptibility against *Staphylococcus* spp. isolated from patients with neutropenic cancer. The E-test was found to be a reliable alternative methodology to the standard test for determining the level of the levofloxacin resistance in staphylococci [34].

Gianecin et al. [36] compared the disk diffusion and agar dilution to study the antimicrobial activity of gentamicin on clinical isolates of *Neisseria gonorrhoeae*. The results indicated that the disk diffusion assay could be an acceptable method for the susceptibility of gentamicin against *Neisseria gonorrhoeae* [36].

**Figure 4:**
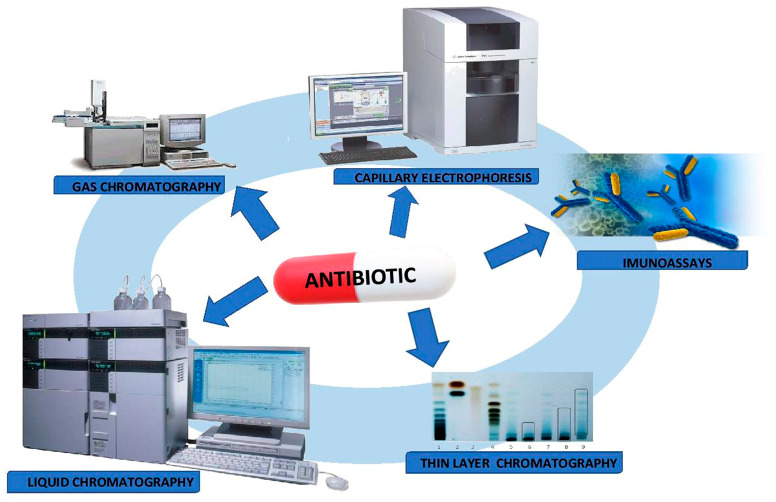
The analytical techniques for determination of antibiotics.

In routine clinical management, the interpretation of the obtained drug concentration measurement requires the following conditions to be met: The knowledge of pharmacokinetics of the drug being tested, existence of a certain correlation between the drug concentration in blood and its therapeutic or toxic effects, determination of the range of therapeutic concentration of the drug being tested, as well as the development of sensitive and specific analytical methods (Figure 4) allowing to determine the drug concentration in body fluids [37,38,39,40].

### 5.2. Analytical Techniques

High-performance liquid chromatography (HPLC) is one of the most commonly used analytical methods for the quantification and qualification analysis of antibiotics in biological samples (plasma, serum, whole blood, urine). In addition to these techniques, determinations by immunochemical tests, gas chromatography (GC), thin-layer liquid chromatography (TLC) or capillary electrophoresis (CE) are also available.

#### 5.2.1. Immunoassays

Immunoassays are analytical methods that enable the detection of substances in clinical samples by creating a stable complex between the analyte and a specific antibody. Antigen-antibody reactions are stoichiometric; therefore, the determination of free or bound antigens leads to a direct calculation of the antibiotic level. However, the disadvantage of immunoassays is their potential lack of specificity, which may lead to cross-reactivity with metabolites, drugs or structurally related compounds [41,42].

Pastor-Navarro et al. [43] used the immunoassay to determine in human plasma the concentration of sulfasalazine. The enzyme-linked immunosorbent assay (ELISA) allowed for the detection of antibiotics at the concentration levels of 0.02 ng/mL [43].

The fluorescence polarization immunoassay (FPIA) assay of levofloxacin in urine was also described by Shanin et al. [44]. The achieved LOD value was 0.5 ng/mL [44].

Dijkstra and co-workers [45] also demonstrated the use of the tobramycin immunoassay kit for the detection of the kanamycin concentration in the serum. The results of the immunoassay method were compared with the LC-MS/MS analysis. This method is able to quantify a large range of kanamycin concentrations in a reliable and reproducible manner [45].

Merola et al. [46] presented an immunosensor technique for the determination of β-lactam antibiotic drugs in the human serum and urine. This technique showed to be very sensitive, cheap and reproducible; the LOD value was about 10^−11^ M [46].

Furthermore, the detection of antibiotic drugs from different groups based on the electrochemical Immunosensor was demonstrated in the review article by Pollap and Kochana in detail [47].

#### 5.2.2. Chromatographic Techniques

In recent years, several rapid, sensitive and specific analytical methods for the determination of the antibiotic content in complex biological matrices have been applied in routine laboratories. These techniques are essential to provide reproducible results that can be used in clinical trials to improve the effectiveness of the antibiotic therapy. The variability of the separation mechanisms enables the identification and determination of antibiotics from different groups, including penicillins, macrolides, aminoglycosides, tetracyclines, quinolones and nitroimidazoles. The choice of the separation method is based on the properties of the analyzed antimicrobial substances, e.g., solubility in water and organic solvents or acid-base properties.

##### Thin Layer Chromatography (TLC)

According to the WHO European Pharmacopoeia [48], the use of liquid thin layer chromatography (TLC) is recommended for the identification of specific antibiotics (neomycin). Jain et al. [49] separated minocycline in plasma using the TLC gel coated with silica 60F254 and sprayed with a mixture of methanol-acetonitrile-isopropanol-water (5:4:0.5:0.5 (*v*/*v*)). The antibiotics were identified by the UV irradiation at 190–400 nm wavelengths. The accuracy of the method expressed as the percentage of recovery was from 95.08% to 100.6%. The method meets the acceptance criteria for validation and may be useful for the determination of minocycline in the human plasma [49]. The HPTLC method with densitometric detection for the determination of amoxicillin and ampicillin in urine samples was described by Gholipour et al. [50]. Separation was effected on titanium(IV) silicate TLC plates using a mixture of mobile phase (K_2_HPO_4_ (0.1 M) + KH_2_PO_4_ (0.1 M), 1:1 (*v*/*v*)), and the relevant compounds visualized by spraying with 1% solution of ninhydrin in ethanol. The TLC suggested technique provided a simple, accurate, and reproducible analysis of both amoxicillin and ampicillin in biological samples [50]. Unfortunately, due to the relatively low repeatability and difficult validation of results, the TLC method is not preferred for quantitative determinations.

An alternative approach consists in combining directly planar chromatography with mass spectrometry. In particular, matrix-assisted laser desorption/ionization (MALDI-TOF/MS) is a modern ionization technique that can be combined with thin layer chromatography (TLC-MALDI-TOF MS) [51].

The main positive aspects of TLC-MALDI-TOF MS were presented in the analysis of the mixture of tetracycline antibiotics. Particles of various materials (Co, TiN, TiO_2_, graphite, silicon) were investigated by using suspensions of particles on eluted TLC plates. Dichloromethane, methanol and water (59:35:8, *v*/*v*/*v*) were applied ss the solvent system. Mass spectra and mass chromatograms were obtained from direct TLC plates. Before the MALDI analysis, only an unresolved spot for tetracycline and chlortetracycline were found in the TLC plate. However, the MALDI mass spectra and the graphing of individual ion chromatograms resulted in separate peaks for chlortetracycline and tetracycline. The TLC-MALDI-TOF MS analysis of tetracyclines enabled the calculation of the R_f_ value of the analyte spots, which indicates good compliance with the retention factor value acquired by using the UV detection [52]

##### Gas Chromatography (GC)

Besides thin layer chromatography, gas chromatography (GC) is also used for the determination of antibiotics in biological fluids. However, the GC technique is very seldom used due to the need to transform drugs and their metabolites into thermostable derivatives [53,54].

Thangadurai [55] described gas chromatography with the mass spectrometric (GC/MS) detection method to determine azithromycin in biological samples (gastric cleavage samples). The sample was extracted with chloroform and cleaned up by n-hexane washing. Then, the cleaned-up extract was acetylated in the acetic anhydride-pyridine mixture (1:2). Azithromycin was analyzed by GC without derivatization. The authors used the phenylmethyl silicone bonded phase GC capillary column (0.25 µm, 30 m × 0.25 mm i.d.). The obtained detection limit was 2 μg/mL^−1^. This method can be used to monitor the antibiotic level in biological materials for forensic and toxicological aims [55].

Chiavarino et al. [56] reported the GC method with the atomic emission detector (GC-AED) for the detection of nine suflonamides. The samples were derivatized using N-methylation. Gas chromatographic separations were achieved on 12.5 m × 0.22 mm i.d. phenylmethyl silicone column. This technique displayed linearity and may be used for the quantitative determination of suflonamides [56].

##### Liquid Chromatography (LC)

However, high-performance liquid chromatography (HPLC) plays an important role in the determination of antibacterial drugs in body fluids. A wide spectrum of detectors used for determination (UV, DAD, PDA, FL, MS as well as universal detectors: CAD or ELSD) and modern methods of sample preparation for the analysis enabled to obtain reproducible results, even in complex matrices [57,58,59,60,61,62,63,64,65,66,67]. Nonetheless, LC-MS/MS is the only technique that ensures unambiguous analysis.

Borner et al. [66] developed an HPLC assay for the determination of linezolid in human plasma and urine using a Nucleosil-100 5C18 column. The mobile phase was composed of acetonitrile/sodium acetate buffer/water 18/10/72 *v*/*v*. The elution of drugs was monitored at 250 nm. This paper addressed the compatibility of the results obtained using microbiological tests and the HPLC method in respect of serum and urine [66]. In 2009, Farshchi, Ghiasi and Bahram [68] described an HPLC protocol for the analysis of clarithromycin in the human serum after derivatization with 9-fluorenylmethyl chloroformate (FMOC-Cl). Following the liquid-liquid extraction (dichloromethane) of the antibiotic, the compounds were analyzed using HPLC with a fluorescence detector (HPLC-FL). The HPLC column used for the analysis was 150 cm long with a 4.6 mm internal diameter and a particle size of 5 µm. The authors concluded that the analysis time was reduced, the LOQ value was enhanced and the time needed for the derivatization of the clarithromycin in the human serum was also shortened [68]. Locatelli [60] and his co-researchers reported a rapid HPLC assay with the microextraction for the analysis of two fluoroquinolones (ciprofloxacin and levofloxacin) in the human sputum. Chromatographic separation was achieved by using a Gemini C18 column (250 mm × 4.6 mm i.d., 5 µm) and mobile phase was composed of a mixture of phosphate buffer (30 mM, pH 2.5, 1% triethylamine (TEA)), and acetonitrile (1% TEA) (86:14, *v*:*v*) at 1.0 mL/min flow rate. The detection of peaks was achieved by the photodiode array detector (PDA) at 295 nm for levofloxacin and at 279 nm for ciprofloxacin. The research suggested that MEPS-HPLC-PDA in off-line mode was an effective method for the quantitative determination of ciprofloxacin and levofloxacin in clinical specimens [60].

Buszewski et al. [61] described a sensitive method for the determination of five antibiotics and their metabolites in the whole blood and tissues. The analysis was carried out using the HPLC combined with tandem mass spectrometry. After the solid phase microextraction (SPME), the specimen was determined using an analytical C18 column (50 mm × 2.0 mm i.d., 4 µm) and a mobile phase consisted of water (0.1% formic acid) and acetonitrile. Detection was achieved by a triple-quadrupole mass spectrometer (HPLC-QqQ-MS) with an electrospray ionization (ESI). This is an important finding in the identification of antibiotics (amoxicillin, cefotaxime, ciprofloxacin, clindamycin and metronidazole) and their metabolites in the biological matrix using the SPME sample preparation technique. Moreover, two mass spectrometric techniques: ESI-QqQ and MALDI TOF, were demonstrated to be complementary in the determination of active compounds in clinical samples [61].

Ultra-performance liquid chromatography with tandem mass spectrometry (UPLC-MS/MS) of seven antibiotics in human serum was also reported [69]. After protein precipitation, (ACN) drugs were separated by using the UPLC HSS T3 column (100 mm, 2.1 mm i.d., 1.8 µm) and a mixture of mobile phase: 5 mM ammonium acetate (pH 2.45) and acetonitrile. The authors achieved a quantification lower limit (LLOQ) of 0.1 µg/mL. In conclusion, the UPLC-MS/MS method seems to improve the limit of quantification and shorten the analysis time. The authors suggested that the proposed method is simple, fast, sensitive and suitable for clinical studies particularly in neonate patients [69].

It is also noteworthy to analyze polar drugs by hydrophilic interaction liquid chromatography (HILIC). Kathriarachchi [70] et al. performed separations of amoxicillin and metronidazole in the human serum using the HILIC technique. The chromatographic separation was obtained on the ZIC-HILIC column and the mobile phase included 0.1% (*v*/*v*) formic acid in water and 0.1% (*v*/*v*) formic acid in acetonitrile. The method was fully validated and the lowest limit of quantification was 0.0138 μg/mL for amoxicillin and 0.008 μg/mL for metronidazole. The linearity was from 0.1 μg/mL to 6.4 μg/mL for both antibiotic drugs [70].

Other examples of the determination and identification of antibiotics by liquid chromatography in different biological fluids are summarized in Table 2. 

##### Electromigration Techniques

**Figure 5:**
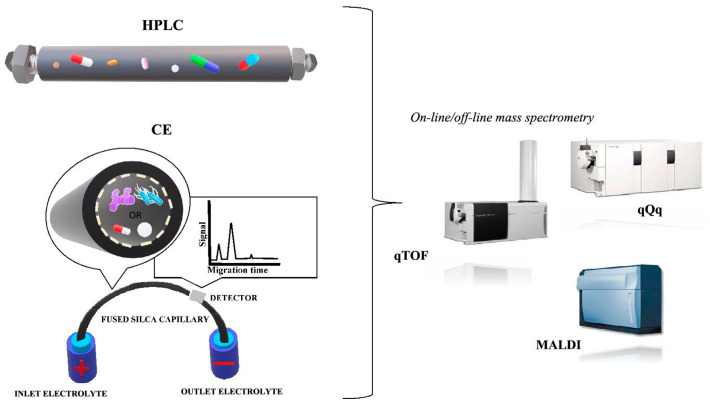
A comparison of the separation analysis by high performance liquid chromatography (HPLC) and capillary electrophoresis (CE).

Electromigration techniques are also separation analytical techniques used to measure various drugs, including antibiotics, especially for polar drugs and stereoisomer analysis. Electrokinetic analyses are based on electrokinetic phenomena: electromigration of ions, charged particles and electroosmosis. These phenomena appear in solutions when charged particles are placed in an electric field, mainly with high voltage. A comparison of the separation of analytes by the capillary electrophoresis and liquid chromatography is presented in Figure 5. Depending on the separation mechanism, we can distinguish between capillary zone electrophoresis (CZE), micellar capillary electrokinetic chromatography (MEKC), capillary non-aqueous electrophoresis (NACK) and capillary isotachophoresis (CITP). The antibiotic study by capillary electrophoresis mainly includes two modes CZE as well as MEKC. A significant advantage of CE is its availability, simplicity of equipment, the use of small concentrations of organic solvents in the buffer and, above all, a short time of the analysis and high efficiency of the analytic separation.

Most of the proposed methods for the electrophoretic separation of antibiotics in different matrices are based on the use of different detection methods including spectrophotometry (UV), combined with a diode array (DAD), fluorescence (FD), electrochemical detection (ECD) or laser-induced fluorescence (LIF) [83,84,85,86]. In addition, other and more innovative detection methods have recently been used, including such methods as non-contact conductivity detection (C4D) [85,87] or potential gradient detection (PGD) [88].

Solangi et al. [89] used capillary zone electrophoresis (CZE) for the determination of two cephalosporins (cefradine and cefuroxime) in urine. The authors used 42 µm filter paper to filter these drugs and the UV detection at 214 nm. The analysis was performed at 30 kV and 25 °C using 50 mM sodium borate buffer (pH 9). The limits of the detection of two cephalosporin were from 29.0 to 30.2 μg/mL at the recovery 99–100% for cefuroxime and 1.3–1.9% for cefradine [89].

In 2015, a method of coupling CEC with mass spectrometry was also described. Hernández-Mesa et al. [90] determined five nitroimidazoles in urine samples. After SPE, drugs were analyzed by using a column packed with a mixture of Bidentate C18:Lichrospher Silica-60 (5 µm) and the background electrolyte (BGA) composed by acetonitrile, methanol and water (45:10:45 *v*/*v*/*v*). The limit of the detection of the assay was from 0.09 to 0.42 µg/mL [90]. Another report described the CE determination of ceftazidime in human plasma using a capillary column (31.5 cm × 25 µm) and 50 mM chloroacetic acid with 20% *v*/*v* methanol and 0.5% *v*/*v* coating solution of INST. The samples were deproteinized by acetonitrile. The analysis was carried out using 30 kV at 25 °C. The proper identification of ceftazidime in clinical samples constitutes an important aspect in improving the treatment of the diabetic foot [91].

Other examples of the determination and identification and determination of antibiotics and their metabolites by electrophoretic method are summarized in Table 3.

## 6. Different Analytical Techniques for the Determination and Identification of Microorganisms

The detection and identification of the microorganism responsible for the infection is also an essential factor in the implementation of appropriate antibiotic therapy. The changing epidemiology of infections, the emergence of new pathogenic bacteria and the easy spread of pathogens, including drug-resistant strains, make it necessary to improve the existing ones and to search for and develop new methods of microbial identification.

**Figure 6:**
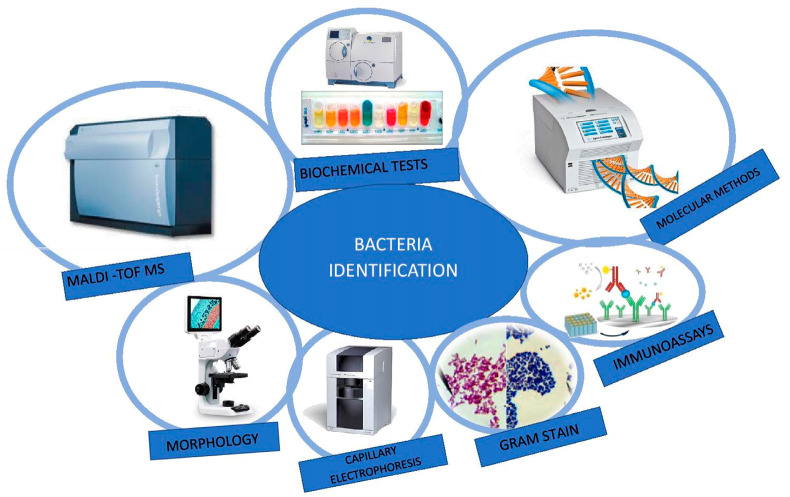
The popular methods to microorganism identification.

Identification is done by matching characteristics (phenotypic or genotypic) to a fixed reference organism such as the strain type. There are a number of standard methods for the detection and identification of pathogenic bacteria (Figure 6). Phenotyping methods allow the microbiologist to identify the microorganism to genus and sometimes species level based on a relatively small number of observations and tests. These methods include biotyping, serotyping, bacteriophage typing, evaluation of susceptibility profiles and protein analysis methods. Biotyping examines biochemical requirements, environmental conditions (pH, temperature, antibiotic resistance, susceptibility to bacteriocins) and physiological aspects (colony and cell morphology, cell walls and cell membrane composition such as fatty acid profiles) [100,101,102].

### 6.1. Gram Staining

A bacterial cell is an organism with a dynamic metabolism, heterogeneous in terms of structural and chemical characteristics. Microorganisms are biochemical reactors with the ability to rapidly assimilate to the environment, undergo mutation and change fast. The cell surface of bacteria consists of several components (e.g., surface proteins) forming an adherent, cohesive layer on the cell surface and affecting the physicochemical properties of the intact microbial cell. Bacteria cells can be differentiated on the basis of the properties of substances bonded to their surface.

**Figure 7:**
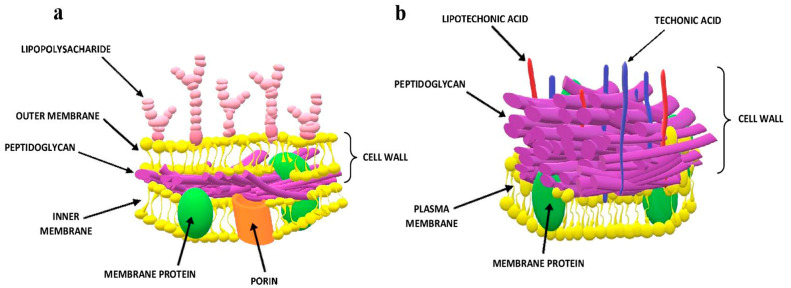
Differences between Gram-negative (**a**) and Gram-positive (**b**) of bacteria cell wall.

Moreover, traditional methods of the microbial identification require the recognition of differences in morphology, growth, enzymatic activity and metabolism to identify the genera and species of bacteria. Colony morphology is usually described by a direct observation of the characteristic features of a colony—size, color, shape. The Gram staining method differentiates bacteria into two groups: violet-colored Gram-positive bacteria and pink-colored Gram-negative bacteria. Color differences result from the difference in the structure of the cell wall of both groups of bacteria (Figure 7). Gram-positive bacteria have a cell wall consisting of a thick layer of peptidoglycan, peptide bridges and lipoteichoic acid molecules (LTA). The cell wall of Gram-negative bacteria has a more complex structure. It consists of a thin layer of peptidoglycan and an outer membrane, connected by bridges formed from the lipoprotein. The peptidoglycan and the outer membrane are separated by the so-called periplasmic space. The outer membrane with the structure of a typical protein-lipid membrane in the outer lipid layer contains lipopolysaccharide (LPS) with the composition characteristic for particular Gram-negative bacteria species. The permeation of the substance through the outer membrane is possible due to the presence of porin protein channels. In most cases, these studies are only the first stage of microbial identification, guiding the subsequent stages of microbiological investigation [103,104].

The Becerra’s group [105] optimized the Gram staining procedure by comparing commonly used Gram stains and collagen counterstain. Gram staining can be a useful tool in the identification of the bacteria species such as *Pseudomonas aeruginosa* and *Staphylococcus aureus* from clinical samples [105]. Bishop et al. [106] used the Gram staining method to determine the bacteria isolated from cerebrospinal fluid samples. They suggested that, in some cases, Gram staining provided sufficient information to start the appropriate antibiotic therapy [106].

### 6.2. Biochemical Tests

A further acceleration of the time of the detection and identification of microorganisms was possible thanks to the introduction of a new generation of automatic systems for the identification of bacteria based on biochemical properties. The identification of bacterial culture based on the principle of comparing the biochemical reaction profile with the database is most often performed using automated sets such as Analytical Profile Index API^®^, BD Phoenix™ Automated Microbiology System or Vitek 2 Compact [100,104]. Commercial automated systems for the identification and determination of bacterial susceptibility are usually based on the same principles as conventional tests, as they use miniaturized versions of these tests. In addition, automated systems not only determine susceptibility but also indicate the likely mechanism of antibiotic resistance, such as extended-spectrum beta-lactamases (ESBL), methicillin resistance in *Staphylococci*, glycopeptide resistance or high-degree aminoglycosides resistance in Enterococci [100,107,108].

Kierzowska and coworkers [109] described the application of the Api 20A system for the determination of various bacteria species isolated from swabs, biopsies, fluids, tissues and pus. The authors concluded that the applied biochemical test fails to give reliable results for the identification of anaerobic bacteria. Moreover, the method is time-consuming and costly [109]. Hogan et al. [110] described the use of the biochemical test, Vitek 2, to assess the susceptibility of the cultured bacterial strains to selected antibiotics. The applied method can be used as a diagnostic tool to estimate the susceptibility of antimicrobial agents to the presence of batteries, especially of the genus *Enterobacteriaceae* [110].

In comparison to traditional diagnostic methods, automated biochemical tests have more advantages. A great advantage is the possibility of a simultaneous identification and determination of the drug susceptibility of the tested microorganism (combo panels in MicroScan^®^ WalkAway and Phoenix™ BD tests). Thanks to the use of biochemical tests, the identification time was significantly shortened. Now, instead of the few days which were needed earlier, the result is obtained within a few hours. In addition to a significant reduction in the time of the identification of the microorganism, an undoubted advantage of these systems is the ability to perform the parallel identification and/or drug susceptibility determinations for many strains of bacteria at the same time. Sensitive detection systems used in the cameras detect even the smallest, subtle changes in the growth of microorganisms, which ensures the precision of the results read out automatically. Moreover, advanced software with modern automated systems allows different ways of generating, processing, collecting and transmitting results. Unfortunately, in biochemical tests, the concentration and uniformity of suspension required for inoculation plays a very critical role in the accuracy of the identification [111,112,113].

The biochemical properties of proliferating microorganisms are also determined using media enriched with one or more chromogenic substrates. The inclusion of such substrates in a selective or non-selective primary medium may significantly shorten the diagnostic procedure, as cultures, isolation and identification are carried out on the same medium. Chromogenic media are widely used for screening to identify patients with antibiotic resistant bacteria such as *S. aureus* resistant to methicillin (MRSA) or vancomycin-Enterokocci (VRE). Chromogenic compounds metabolized by bacteria or fungi of certain species give colonies their characteristic color. Chromogenic substrates used in such media are usually targeted at bacterial hydrolysis—most commonly, glycosidases such as β-galactosidase or β-glucosidase. Other less frequently chosen hydrolyses are esterases or peptidases. For example, the detection of β-alanine aminopeptidase was used to detect *Pseudomonas aeruginosa*. Chromogenic media are offered by many manufacturers, including bioMérieux (chromID media), Merck (Chromocult and Fluorocult media), Bio-Rad Laboratories (Select media). Adding antibiotics to such media enables screening to detect bacteria resistant to antibiotics colonized in the respiratory tract or gastrointestinal tract of patients [107,114,115,116].

### 6.3. Immunoassays

Some *Streptococcus* species contain a unique carbohydrate molecule as part of the cell wall that can be used to distinguish them from other species. Such differences between species can be identified by the use of serological typing. Serotyping is one of the oldest immunological techniques. It is an important method of identification not only for Gram-negative bacteria such as *E. coli* and *Salmonella* spp. but also for some Gram-positive bacteria. Immunological methods use a reaction of bacterial antigens with antibodies against these antigens. Different antibody markers are used to visualize the immune response. In the fluorescence microscopy method, the marker is the fluorescence dye. In the case of latex agglutination, antibodies are coated on latex molecules. In immunoenzymatic methods, however, antibodies or antigens, depending on the variant of the method, are labelled with an enzyme. In diagnostic laboratories, fluorescence immunoanalysis and enzyme linked immunosorbent assay (ELISA) tests are commonly used, in which determinations are performed using various methods of detecting the immune response. Due to the use of antibodies for specific antigens, immunological methods allow to confirm or exclude the presence of only the desired microorganisms [117,118].

### 6.4. Bacteriophage Typing

Another method of phenotyping is bacteriophage typing. Bacteriophage viruses can infect host cells, causing the disintegration or incorporation of their genetic material and the expression of new proteins. These methods can be used in both single and mixed cultures where host specificity allows both detection and identification. These techniques are mainly used for research purposes and their commercial development is primarily intended for use in clinical and food microbiology [119].

### 6.5. Fatty Acid Profile

A more common method of bacterial identification is to characterize the types and proportions of fatty acids present in the cytoplasmic and outer membranes of bacteria. The fatty acid composition of prokaryotes can be very variable and concern the length, presence or absence of a double bond, ring or chain branching. The wealth of information contained in these compounds concerns both qualitative differences (usually at genus level) and quantitative differences (often at species level). Branched chain fatty acids are common in many Gram-positive bacteria, while Gram-negative bacteria consist mainly of simple chain fatty acids. The identification of bacteria based on fatty acid composites (profile) is widely used in clinical laboratories, public health, food and water inspection, where pathogens and other bacterial hazards must be identified routinely [120,121].

Unfortunately, these phenotypic methods are limited because microorganisms can suddenly change their phenotypic properties due to environmental changes or genetic mutations. Therefore, in order to avoid problems that may arise with phenotypic methods, identification on the basis of genotypic traits was developed. These methods include the DNA hybridization, polymerase chain reaction (PCR), rRNA 16s and 23s gene sequencing and fingerprinting (ribotyping) [100,122].

### 6.6. Molecular Methods

#### 6.6.1. DNA Hybridization

The hybridization of nucleic acids involves the formation of hydrogen bonds between nucleotides of complementary single-stranded DNA or RNA molecules. Hybridization results in double-stranded molecules (hybrids) in which one thread is a DNA or RNA molecule of the tested microorganism (target) and the other is chemically, radioactively or fluorescently marked with a probe. DNA, RNA or nucleic acid molecules are used as probes. In microbiological diagnostics, the methods of solid and liquid hybridization are used. Examples of solid hybridization are Southern blot (detection of DNA acid) and Northern blot (detection of RNA acid). One of the types of hybridization is FISH (Fluorescent In Situ Hybridization), which enables the detection of a specific DNA sequence in a tested sample using a molecular probe marked fluorescently. Currently, in microbiological laboratories, the most frequently used technique is hybridization in solution. In commercial systems, labelled probes are used to detect and quickly identify the microorganism that causes the infection, while microbial detection is based on chemiluminescence or fluorescence. Examples of using the hybridization method in microbiological diagnostics are tests: AccuProbe from Gen-Prob Inc., QuickFISH and PNA FISH tests from AdvanDx [122,123].

The identification of microorganisms is increasingly carried out using hybridization with the DNA microarrays (DNA chips) technology. The marked sample (the studied microbiological material) is placed on a plate containing a DNA probe with a known nucleotide sequence. The most common probe sequences are selected from databases such as GeneBank or UniGene. Then the plate is scanned which results in a different intensity of light points, which is caused by the presence of characteristic probes for specific genes. Next, a number of fluorescence intensities are assigned to each point. The data obtained are subjected to the bioinformatic analysis. Microarrays are available in two types: oligonucleotide chip (DNA chip) and cDNA, differing in the size of the nucleic acid [124].

Jin et al. [125] used the oligonucleotide microarrays method to detect intestinal bacteria in fecal samples. The probes were projected on the base of 16S and 23 rRNA gene sequences of 15 intestinal bacteria species. The genes were amplified with two universal primers, and 22 oligonucleotide probes were used for detection. It was demonstrated that the use of the DNA microarray allows for a specific identification of bacteria species dominating in the intestinal microflora [125].

#### 6.6.2. PCR-Based Methods

The introduction of Polymerase Chain Reaction (PCR) was one of the biggest, if not the biggest, breakthroughs in biological and chemical sciences. This method was developed by Mullis et al. [126] in the early 1980s. The technique consists in a multiple duplication of any DNA sequence using temperature-resistant polymerase and primers, i.e., short DNA chains with sequences complementary to the final synthesized DNA fragment sequences. In order to visualize the expected size and purity of the DNA molecule, the reaction product is subjected to electrophoresis in agarose gel and visualized with a DNA binding dye, e.g., ethidium bromide. For identification purposes, the 16S rRNA gene is a beneficial target for PCR amplification as it is widely distributed among bacteria and contains sufficient differences between strains and species in the DNA sequence. Microorganisms can be identified by comparing the 16S rRNA gene sequences available in databases with those of an unknown microorganism [127].

Kouidhi et al. [128] used the DNA amplification to detect typical bacteria present in the oral cavity of children with caries. *Streptococcus mutans*, *Candida albicans*, *Streptococcus salivarius* and *Streptococcus oralis* were identified in most saliva samples of children affected by tooth decay. The authors, therefore, suggested that this method may be useful in monitoring the presence of caries-specific pathogens in the oral cavity [128]. Pechorsky and coworkers [129] showed that PCR methods can be used to identify the pathogenic bacteria from blood matrices. The proposed identification method can provide useful information for the determination of blood stains in clinical laboratories [129].

Despite the development of various molecular methods, PCR remains the most widely used method, both in experimental research and in clinical laboratories. This method is often used to simultaneously detect both the PCR positive control DNA and the tested DNA in a single tube, or two different target sequences in the tested DNA. A variation of the PCR method is real-time PCR (RT-PCR), carried out in special apparatus and with appropriately prepared starters. It allows to read the result of the reaction during its course, by measuring the fluorescence of the sample, which is proportional to the amount of the product produced. Both classic and RT-PCR methods are used to determine the presence of microorganisms in bacteria, viruses and fungi. Currently, there are various PCR systems available on the market which enable the detection of microorganisms directly in the test sample (blood, serum, plasma, cerebrospinal fluid) or the presence of genes, encoding toxins or mechanisms of antibiotic resistance in the cultured bacteria [122]. Examples of such tests are GeneXpert from Cepheid, which enables the detection of e.g., MRSA in nasal swabs, positive blood culture bottle samples and wound swabs, and vancomycin-resistant enterococci (VRE) in rectal swabs [130]. The disadvantage of PCR systems is targeted testing, which means that we confirm or exclude the presence of specific microorganisms. In addition, 16S analyses of the rRNA gene sequences showed limited variability within bacterial strains such as *Bacillus cereus*. Therefore, due to high homology, this technique is not always reliable in the identification of an unknown organism [131].

PCR/ESI-MS is another microbial identification method using a combination of molecular biology techniques and mass spectrometry. Many starter pairs are used for PCR: Starters specific to entire groups of microorganisms, starters specific to species or strain, and starters aimed at antibiotic resistance genes or genes responsible for pathogenicity. After receiving PCR products, molecular masses of the DNA fragments obtained are determined using ESI-MS. The results of molecular mass determination of amplicons are species-specific code—”fingerprint”, which is compared with the results stored in the database [121,127,132]. The study by Brinkman et al. [133] showed that ESI-MS PCR technology can be a useful tool in the treatment of infectious endocarditis [133].

Modern microbiological analysis is usually carried out by traditional cultures and molecular biology techniques such as PCR. However, the problem with using conventional microbiological techniques is that they are time-consuming and costly. In addition, information obtained from these tests does not provide any insight into the molecular profile of bacteria and protein expression induced by the stress factor. Therefore, an innovative analytical approach was developed, based on the electrophoretic (CZE) [134,135,136,137,138] and spectral analysis of microorganisms, using matrix-assisted laser desorption/ionization with time of flight (MALDI-TOF) [139,140,141].

### 6.7. Matrix-Assisted Laser Desorption/Ionization Time-of-Flight Mass Spectrometry (MALDI-TOF MS)

**Figure 8:**
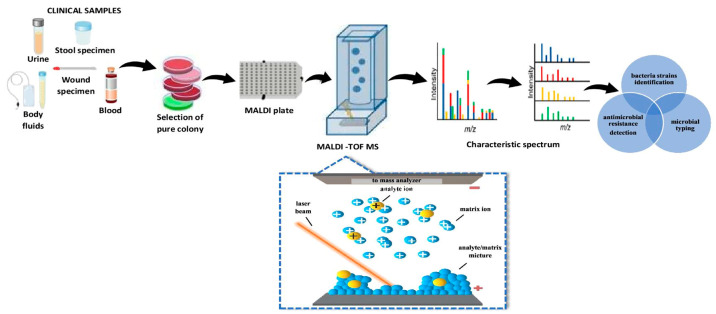
Schematic illustration of MALDI-TOF MS (matrix-assisted laser desorption/ionization with time of flight) analysis.

Recently, we have also used devices that allow us to identify microorganisms based on the analysis of protein profiles. Innovative mass spectrometry technology, or rather a variant of this technique, abbreviated as the MALDI-TOF, is used more and more commonly in microbiological diagnostics. In this method, the sample is subjected to a matrix that absorbs energy from the laser, resulting in the rapid heating, evaporation and ionization of the analytes; the ions are then separated based on their time taken to reach the detector, as all ions of the same charge receive the same kinetic energy highly abundant proteins, then the ribosomal proteins are analyzed [100,142,143,144]. Figure 8 shows a schematic diagram of the MALDI-TOF MS analysis [134,136].

The MALDI-TOF MS technique is recommended mainly for biochemical and clinical analysis; however, the absence of an accurate database limits its technological capabilities and benefits [141,142,143,144,145]. The potential of the MALDI-TOF MS makes it possible to extend the applications to other fields of microbiological analysis, pharmacology, food technology or environmental analysis. Therefore, an early detection of the pathogen will facilitate an appropriate preventive action (dedicated therapy, target analysis). In the case of the medical analysis, this means selecting an appropriate treatment. The biggest drawback of the spectrometric analysis of microorganisms is the scarcity of the databases (repositories). Firstly, the limited number of producers and distributors of bacteria-identifying software results in exaggerated prices of the databases. For this reason, the spectrum of microorganisms contained therein may not coincide with the bacteria which are the subject of our research. Therefore, it is possible to combine the identification of the bacterial strain by the commercial software (Biotyper, Bruker Daltonics) with the development of cheap, fast and precise reference repositories of bacterial spectra [140,146,147,148,149]. Furthermore, coupling of molecular biology techniques with traditional culture methods in developing of the data needed to create repositories is an interdisciplinary approach to the problem of the credibility of reference strains. Another problem is related to drug resistance mechanisms and antiseptic sensitivity. As drugs are now universally applied, the response of human immunological systems to bacterial, yeast and viral infections is getting weaker and weaker [150]. The result is the phenomenon of drug resistance in bacteria. A detailed study of the problem constitutes a modern analytical challenge.

The generated spectrum of peaks corresponding to ions of different mass to charge ratio corresponds to a unique protein profile, a specific molecular “fingerprint” of the tested microorganism. The spectrum is compared with the spectra of reference microorganisms collected in the database. The probability of correct identification is expressed by a point indicator which, depending on the value obtained, indicates a reliable identification of the micro-organism to the species reliable identification to the genus level with a probable identification to the species or the probable result of identification to the genus level. When the value of an indicator is below the accepted value, there is no reliable identification result. In microbiological laboratories, we can see systems such as Bruker’s MALDI BioTyper or VITEK^®^ MS from bioMérieux. These systems deliver results in minutes. Currently, the MALDI-TOF MS is mainly used in culture methods to confirm the identification of bacteria [146].

Mailhac et al. [151] described the use of this method to identify 45 bacterial isolates from vitreous samples. Moreover, the authors optimized the protocol for the extraction of bacterial protein. The study showed that 96% of bacterial isolates were identified by species [151]. Haiko et al. [152] demonstrated that MALDI-TOF MS can also be a diagnostic tool for determining the bacteria responsible for urinary tract infections. From 107 Gram-negative bacterial isolates tested, the MALDI-TOF MS method identified 92 (86%) of them. MALDI-TOF MS is a valuable method for a rapid diagnostic of pathogens in patients with urinary infections [152].

In addition, the MALDI-TOF MS applications in clinical microbiology go beyond the identification of microorganisms, and this technique can also be used for the rapid detection of antimicrobial resistance. It was observed that products that result from hydrolysis (e.g., β-lactams by bacterial enzymes—β-lactamases) differ in molecular weight from native antibiotic molecules. There are also studies that indicate the enormous potential of this method in a routine detection of dangerous resistance mechanisms, e.g., carbapenemase. Despite the success, there are some limitations in the use of the MALDI-TOF MS, such as the inability to determine taxonomically related bacteria, e.g., highly pathogenic *Shigella* species from commensal *Escherichia coli*, and the inability to identify *Streptococcus pneumoniae* from some commensal oral *Streptococci* species. Yet, these systems are still being improved and their sensitivity will probably increase with each next generation, which shall strengthen the position of spectrometric mass in clinical laboratories [153,154]. In Table 4, the identification of bacteria in clinical samples by using the MALDI-TOF MS technique is presented.

### 6.8. Capillary Electrophoresis

Moreover, capillary electrophoresis has also been developed over the past decade. Although these newer methods will not replace the traditional methods of plate counting involving cultures and microscopes, their development and use will not expand further. Like other colloidal particles, the microorganism transfers the charged groups to its outer surface, and their electric double layer is created when the charged microorganism comes into contact with aqueous solution (BGE, background electrolyte). Therefore, under the influence of an electric field, bacteria show a characteristic electrophoretic mobility, which is a function of the size of the microorganism, its surface charge and the double electric layer. As we know, capillary electrophoresis quickly and effectively separates biologically important molecules such as proteins and nucleotides. These advantages can also be used for the microbial analysis, as CE methods allow a rapid and simultaneous analysis of several microorganisms in a single sample, including their identification and also quantification [164,165].

The first reports of using capillary electrophoresis for bacteria determination were published in 1987 by Hjerten et al. [166]. They described the migration of Mosaic tobacco virus and *Lactobacillus casei* bacteria in 20 mM buffer Tris-HCl (pH = 7.5); however, they did not achieve any separation. The bacteria migrated along with the electroosmotic flow and acted like units with no electric charge on the surface. In 1993, Ebersole and McCormic [167] separated four types of bacteria in TBE buffer at pH = 9.5, and by gathering particular fractions after the process of electrophoresis had been completed, they proved that the majority of bacteria (80%) were alive. The next year, Torimura [168] published his work concerning the electrophoretic behavior of nine types of bacteria, determining their electrophoretic mobility. In the 1990s, Pfetsch and Welsch [169] and Glynn [170] determined the electrophoretic mobility in different buffer solutions and proved its decrease when ionic force was growing. After 1999, Armstrong et al. [171] introduced poly(ethylene)oxide—PEO, previously used in protein separation. Adding PEO to buffer solutions caused the suppression of electroosmotic flow and significantly lowered the adhesion of bacteria cells to the internal capillary surfaces. The same research group proposed three different mechanisms of bacteria migration in the electric field using PEO, and also the creation of agglomerates by bacteria cells [172]. Zeng and Yeung [173] used a CCD camera for the visualization of cell aggregation; they observed that the cells were moving in different directions at different velocities, depending on the agglomeration size.

Buszewski et al. [135,174,175] developed a modern, extremely fast method for identifying pathogenic microorganisms based on electromigration techniques (capillary electrophoresis). In the experiments conducted so far, several bacterial strains were detected and identified, including those which are as dangerous as *Staphylococcus aureus* [174,175] and *Escherichia coli* [135].

The development of an innovative methodology to identify microorganisms, based on a rapid and selective electrophoretic and spectrometric method, can be a very good analytical solution that brings measurable results, such as a reference set and a screening method. The decrease in people’s resistance to pathogens which is caused by a prolonged use of antibiotics, forces a new approach of understanding and combating drug resistance. Thus, a synthesis of a new generation of antibiotics based on metal complexes can create a desirable pharmaceutical product. The application of the MALDI technology and electromigration techniques in a microbiological analysis can become a milestone in the diagnosis and analysis of the infection. Hence, the relevant procedure should be characterized by: (i) simplicity—the equipment needed can be used in any room, and it only requires basic maintenance; (ii) speed—time of the identification of the presence of relevant bacteria in the prescribed conditions and performing the target metabolomics analysis will be no longer than 60 min—compared to the traditional methods, it is a revolutionary speed; (iii) sensitivity and reproducibility—while maintaining stable parameters of the study; thus, it is supposed to be an absolutely reproducible method.

## 7. Conclusions

The monitoring of therapeutic drugs (TDM) provides valuable information on the actual antibiotic concentration in body fluids. Taking into account the clinical, cognitive and diagnostic purposes of drug monitoring, it is important to select an appropriate analytical method that meets all the requirements. One of the first methods of antibiotic determination in biological matrices were immunoenzymatic techniques, which are characterized by their wide determinability, high sensitivity and a short time of analysis, because they do not require separate techniques of isolation from the biological material. Unfortunately, these methods are not free from defects. An important problem in the use of these tests is the non-specificity of the response to the individual antibiotics of the group. Cross-reactions caused by another group of compounds or due to the influence of the biological matrix may also occur. Moreover, an element making it difficult to fully assess the quantitative dependence in the tested material is the frequent phenomenon of combined determination with the parent compound of its metabolites, the presence of which may interfere with the absolute values of the obtained result.

Therefore, chromatographic methods allowing the determination of both the active compound and its metabolites are increasingly used in everyday practice. These methods include liquid chromatography (LC), high-performance liquid chromatography (HPLC), gas chromatography (GC), which are usually coupled with a mass spectrometry detector (MS), and thin-layer liquid chromatography (TLC). The UHPLC-MS/MS method is mentioned in literature as commonly used. The combination of liquid chromatography with a mass spectrometer guarantees high selectivity, high sensitivity, resolution, repeatability, identification by mass and structure determination by fragmentation, along with the versatility of application. Capillary electrophoresis (CE) is used in new analytical methods. Both HPLC and CE are universal methods, commonly used in monitoring the concentration of antibiotics. However, an essential element of achieving reliable results of monitored drugs is their effective extraction from the biological matrix and appropriate selection of parameters of chromatographic separation and detection. In practice, the LLE method and extraction on columns filled with solid media (SPE) are the most frequently used methods. The frequency of the use of both techniques is comparable.

In recent years, clinical microbiology laboratories have experienced revolutionary changes in the way microorganisms are identified. Until now, the identification of microorganisms in clinical microbiology laboratories has been carried out mainly through the analysis of biochemical reactions and phenotypic features such as growth on different media, colony morphology and Gram staining. Combined, these routine laboratory techniques provide accurate identification of most microorganisms but are costly and time-consuming.

The development of micro and nanotechnology also allowed the use of the DNA microarrays in medical diagnostics. The advantage of this method, in comparison with the previous techniques, is their ability to study the expression of a large number of genes at the same time. Moreover, the DNA microarray requires a relatively small amount of genetic material and is highly sensitive. Unfortunately, the main barrier is the high cost of the arrays and the equipment necessary to carry out tests.

However, the MALDI-TOF MS may be an interesting alternative, especially in some areas where a rapid analysis is required, e.g., in clinical microbiology. This method is not targeted, which means that no prior knowledge of the infectious agent is required, since identification is based on a database match. The level of confidence in the match is calculated using an algorithm, thus eliminating errors of human judgment that plague traditional phenotypic analysis methods such as a morphological analysis. Although the purchase of the machine is relatively expensive, the cost per sample is very low, which translates into significant savings in laboratory operating costs. Finally, while the sample preparation stage requires a certain amount of time, the acquisition and matching of the MALDI spectrum itself is achieved in a matter of minutes. As mentioned earlier, the identification of the MALDI-TOF MS is based on the analysis of the protein spectrum of the bacterial ribosome and is therefore closely related to the analysis of the 16S rDNA gene sequence. However, due to the high similarity of these sequences in some species such as *Shigella* spp., *Escherichia coli* or *Streptococcus pneumoniae* and other members of the *Streptococcus* group (*S. mitis, S. vestibularis*), discrepancies in the identification of these species may occur. In this case, standard biochemical tests such as antigen detection or molecular methods are required. Nevertheless, the MALDI-TOF MS continues to evaluate and improve the equipment of the microbiologist’s tools. Other applications of the MALDI-TOF MS in the development and the possibility of detecting bacterial resistance aroused great interest. The detection principle is based on the hydrolysis of the β-lactam ring in the presence of bacterial enzymes. MALDI-TOF MS is also able to detect changes in the mass of antibiotics caused by the chemical modification in an antimicrobial molecule. In addition, the optimization of sample preparation protocols and the increased representation of less common microorganisms in commercial databases promise a faster and more accurate identification of microorganisms, which, as expected, will translate into better patient care.

The application of the MALDI-TOF MS, the electrophoretic approach and qPCR make a comprehensive interpretation and validation of the results possible. A promising alternative is also the use of a bacterial chip that can act as a sensor to detect bacteria from the outside environment. In addition, it can be utilized as a MALDI-TOF MS target plate for a direct detection of bacteria from clinical samples. On the other hand, along with the development of chromatographic techniques and combining them with sensitive methods of detection, the application of metabolomics has largely increased in recent years. It plays a major role in medicine and pharmacy as well as in agriculture. Thanks to it, the identification of compounds in biological samples for the purpose of clinical diagnosing of diseases is now possible. Moreover, in the pharmaceutical analysis, there exists an important trend to determine metabolic profiles after the administration of a drug in order to trace what is happening with it in the organism. Additionally, the clinical significance of pharmacokinetics stems from the need to use a personalized treatment for each patient, as in such cases, the knowledge of the drug concentration in blood and its determined physical and chemical parameters are very useful in setting a scheme of dosage.

## Figures and Tables

**Table 1:** The antibacterial activity of selected antibiotic metabolites.

Antibiotic (Antibiotics Group)	Metabolite	Activity of the Metabolite Compared to Initial Compound	MIC (µg/mL)		**Ref.**
			A	M	
Metronidazole (nitroimidazole)	1-(2-hydroxyethyl)-2-hydroxymethyl-5-nitroimidazole	<*Bacteroides* spp. <*Clostridium perfringens* <*Clostridium* spp. =*Peptococcus* spp.	0.5 0.5 0.5 0.25	1.0 1.0 2.0 0.25	[21]
Metronidazole (nitroimidazole)	2-methyl-5-nitroimidazole-1-acetic acid	<*Bacteroides* spp. <*Clostridium perfringens* <*Clostridium* spp. <*Peptococcus* spp.	0.5 0.5 0.5 0.25	16.0 32.0 16.0 16.0	[21]
Clarithromycin (macrolide)	14-hydroxyclarithromycin	>*Haemophilus influenzae*	2.4	1.2	[22]
Cefotaxime (β-lactam)	desacetylcefotaxime	=*Pseudomonas aeruginosa* <*Escherichia coli* >*Proteus mirabilis* <*Shigella* spp. <*Klebsiella pneumoniae*	>128 0.25 0.5 0.125 0.25	>128 0.5 0.25 1.0 0.5	[23]
Fidaxcomicin (macrolide)	OP-1118	<*Clostridium perfringens* <*Clostridium difficile*	0.008 0.12	0.25 4.0	[24]
Tinidazole (nitroimidazole)	hydroxytinidzole	>*Gardnerella vaginalis*	32	2	[25]
Metronidazole (nitroimidazole)	hydroxymetronidazole	>*Gardnerella vaginalis*	32	4	[25]
norfloxacin (quinolone)	*N*-nitrosonorfloxacin	<*Enterococcus faecalis* <*Escherichia coli* <*Staphylococcus aureus* <*Mycobacterium gilvum * <*Pseudomonas aeruginosa*	3.01 0.05 1.6 6.2 1.6	7.5 1.9 3.8 12.5 7.5	[26]
norfloxacin (quinolone)	*N*-acetylnorfloxacin	<*Enterococcus faecalis* <*Escherichia coli* <*Staphylococcus aureus* <*Mycobacterium gilvum* <*Pseudomonas aeruginosa*	3.01 0.05 1.6 6.2 1.6	≥50 ≥50 ≥50 ≥50 ≥50	[26]
Cefetamet (β-lactam)	cefetamet pivoxil	=*Escherichia coli* =*Streptococcus pyogenes*	0.5 0.06	0.5 0.06	[27]
Ceftiofur (β-lactam)	desfuroylceftiofur	<*Salmonella* spp. <*Actinobacillus pleuropneumoniae*	1.0 0.0078	2.0 0.015	[28]

A, antibiotic; M, metabolite; <, the metabolite indicates lower antimicrobial activity than the parent compound; >, the metabolite indicates higher antimicrobial activity than the parent compound; =, the metabolite indicates comparable antimicrobial activity to the parent compound.

**Table 2:** Identification and determination of antibiotics and their metabolites by the liquid chromatography technique.

Antibiotic/Metabolite	Matrix	Sample Preparation	Detection	Conditions	LOD/LOQ (units)	Ref.
ceftriaxone metronidazole hydroxymetronidazole	human plasma	protein precipitation (ACN)	HPLC-MS/MS *m*/*z* Q1→Q3 555.1→ 396.0 172.2→128.2 188.0→125.9	Column: Polaris 5 C18-A (150 mm × 3.0 mm i.d., 3.0 µm) Mobile phase: 10mM ammonium formate (pH 2.5)/acetonitrile (0.1% FA) gradient elution 300 µL/min, 30 °C, 5 µL	-/0.4–300 µg/mL (ceftriaxone) -/0.05–50 µg/ mL (metronidazole) -/0.02–30 µg/mL (hydroxymetronidazole)	[71]
ceftriaxone	human plasma	protein precipitation (MeOH)	LC-MS/MS *m*/*z* Q1→Q3 555.0→396.1	Column: Agilent Zorbax Eclipse Plus C18 (100 mm × 2.1 mm i.d., 3.5 μm) Mobile phase: 10mM ammonium formate/acetonitrile (2% FA) (87.5:12.5 *v*/*v*) methanol/acetonitrile (75:25 *v*/*v*) 20 mM ammonium bicarbonate gradient elution 0.4 mL/min, 40 °C, 2 µL	-/1.01–200 µg/mL	[65]
amoxicillin ampicillin cloxacillin dicloxacillin	urine	Filtration (0.45 µm)	LC-UV 210 nm	Column: Zorbax C18 (150 mm × 4.6 mm i.d., 5.0 µm) Mobile phase: 0.11M SDS/6% propanol/0.01M NaH_2_PO_4_ buffer (pH 3.0) mL/min, 25 °C, 20 µL	1.5–15/50 ng/mL	[58]
amoxicillin meropenem ceftazidime cefuroxime piperacillin	human plasma	protein precipitation (ACN)	UPLC-MS/MS *m*/*z* Q1→Q3 366.1→114.0 384.2→141.2 547.1→468.0 442.2→364.1 518.2→143.1	Column: Waters Acquity UPLC BEH C18 (100 mm × 2.1 mm i.d., 1.7 µm) Mobile phase: 2 mM ammonium acetate/water (0.1% FA) 2 mM ammonium acetate/methanol (0.1% FA) gradient elution 0.4 mL/min, 50 °C, 40 µL	-/1.0–100 mg/L (amoxicillin, cefuroxime) -/0.5–80 mg/L (meropenem, ceftazidime) -/1.0–150 mg/L (piperacillin)	[72]
cefazolin cefalothin	human plasma urine peritoneal dialysate	protein precipitation (ACN) filtration (0.45 µm) direct injection	UHPLC-MS/MS *m*/*z* Q1→Q3 455.1→323.1 419.1→315.0	Column: Phenomenex Kinetex C8 (50 mm × 2.1 i.d., 1.7 μm) Mobile phase: 0.1% formic acid 0.1% formic acid/methanol gradient elution 50 °C, 0.2 µL	0.04–0.05/1 µg/mL (plasma) 0.46–4.6/0.1–0.2 µg/mL (urine) 0.01–0.03 /0.2 µg/mL (peritoneal dialysate)	[73]
clarithromycin	human serum	LLE (DCM) derivatization (FMOC-Cl)	HPLC-FD 265 nm (Ex) 315 nm (Em)	Column: Shimpack CLC-ODS (150 mm × 4.6 mm i.d., 5 µm) Mobile phase: 0.05 M phosphate buffer/TEA/methanol 2.0 mL/min., 58 °C, 20 µL	0.01/0.025 µg/mL	[68]
metronidazole	human plasma	LLE protein precipitation (ACN)	HPLC-UV 320 nm	Column: Eclipse XDB-phenyl (250 mm × 4.6 mm i.d., 5 µm) Mobile phase: 0.05 M sodium acetate/acetonitrile/glacial acetic acid (75:25:1 *v*/*v*/*v*) (pH 4.0) 50 µL	-/0.05–30 µg/mL	[74]
metronidazole	human feces	LLE (MeOH)	LC-MS/MS *m*/*z* Q1→Q3 172.2→128.0	Column: Waters Acquity UPLC BEH C18 (50 mm × 2.1 mm i.d., 1.7 µm) Mobile phase: 2 mM ammonium acetate/water (0.1% FA) 2 mM ammonium acetate/water (0.1% FA) gradient elution 0.4 mL/min, 55 °C	5/66 ng/mL	[75]
levornidazole hydroxylation metabolite N-dealkylation metabolite oxidative dechlorination metabolite	human feces	LLE protein precipitation (MeOH)	HPLC-MS/MS *m*/*z* Q1→Q3 220.0→128.0 236.0→171.0 202.0→128.0 299.9→128.1	Column: Atlantis T3 columns (150 mm × 2.1 mm i.d., 5.0 µm) Mobile phase: acetonitrile-methanol/water (0.5% FA) gradient elution 0.4 mL/min, 30 °C	-/0.005–2.0 µg/mL	[76]
cefepime meropenem ciprofloxacin moxifloxacin linezolid piperacillin	human serum	protein precipitation (methanol -methyl-tert-butyl ether (90:10, *v*/*v*)	HPLC-MS/MS *m*/*z* Q1→Q3 481.0→167.0 384.1→114.0 332.0→231.0 402.0→261.0 338.0→235.0 518.0→143.0	Column: Fortis C8 (100 mm × 2.1 mm i.d., 3 µm) Mobile phase: 10 mM ammonium formiate/water (0.1% FA) methanol gradient elution 0.5 mL/min, 30 °C, 15 µL	-/0.25–200 mg/L (cefepime) -/0.25–120 mg/L (meropenem, -/0.05–10 mg/L (ciprofloxacin) -/0.125–10 mg/L (moxifloxacin) -/0.125–50 mg/L (linezolid) -/0.5–400 mg/L (piperacillin)	[67]
cycloserine	human plasma	SPE (ACN)	HPLC-PDA 240 nm	Column: Allantis T3 (150 mm × 4.6 mm id, 3 µm) Mobile phase:10Mm phosphate buffer/acetonitrile (95:5 *v*/*v*) 0.4 mL/min, 30 °C, 50 µL	0.3/1.2 µg/mL	[77]
linezolid	human serum urine	dilution (acetate buffer, pH 3.5)	HPLC-UV 250 nm	Column: Nucleosil-100 5C18 (125 mm × 4 mm id, 5 µm) Mobile phase: Acetonitrile/sodium acetate buffer/water (180:100:720, *v*/*v*), (pH 3.7) 1.3 mL/min, 25 °C, 50 µL	0.07/0.14 mg/L (serum) 2.4/4.7 mg/L (urine)	[66]
fosfomycin	human plasma urine	protein precipitation (ACN) filtration (0.22 µm)	LC-MS/MS *m*/*z* Q1→Q2 137.1→78.9	Column: Merck SeQuant zic-HILIC (50 mm × 2.1 mm i.d., 5 µm) Mobile phase: 2 mM ammonium acetate/acetonitrile (15:85 *v*/*v*) 0.3 mL/min, 24 °C, 0.1µl (plasma), 0.5 µL (urine)	0.01/1.02 µg/mL (plasma) 0.01/0.1 mg/mL (urine)	[78]
amoxicillin oxacillin cloxacillin dicloxacillin	plasma whole blood urine	protein precipitation (ACN) SPE (MeOH)	HPLC-PDA 240 nm	Column: Inertsil ODS-3 (250 mm × 4.0 mm i.d., 5 µm) Mobile phase: acetonitrile (0.1% TFA) 1.0 mL/min, 1.3 mL/min, 25 °C, 20 µL	3.3–6.6/10–20 ng/mL (plasma) 6.6/20 ng/mL (whole blood, urine)	[79]
amoxicillin cefotaxime ciprofloxacin clindamycin metronidazole amoxycilloic acid 4-hydroxyphenylglycyl amoxicillin desacetyl cefotaxime 3-desacetyl cefotaxime lactone ciprofloxacin N-oxide *N*-demethylclindamycin clindamycin sulfoxide hydroxymetronidazole	whole blood surgical wound	SPME (MeOH)	HPLC-QqQ-MS *m*/*z* Q1→Q3 366.0→114.0 456.0→396.0 332.0→314.0 425.0→162.0 172.0→128.0 384.0→189.0 515.0→263.0 414.0→354.0 396.0→336.0 348.0→328.0 411.0→148.0 441.0→178.0 188.0→144.0	Column: Phenomex GRACE C18 (50 mm × 2.0 mm i.d., 4 µm) Mobile phase: acetonitrile/water (0.1% FA) gradient elution 0.4 mL/min, 25 °C, 5 µL	0.031/0.093 µg/mL (amoxicillin) 0.033/0.098 µg/mL (amoxycilloic acid) 0.037/0.112 µg/mL (4-hydroxyphenylglycyl amoxicillin) 0.039/0.118 µg/mL (cefotaxime) 0.041/0.123 µg/mL (3-desacetyl cefotaxime lactone) 0.044/0.131 µg/mL (desacetyl cefotaxime) 0.028/0.085 µg/mL (ciprofloxacin) 0.032/0.096 µg/mL (ciprofloxacin N-oxide) 0.033/0.098 µg/mL (clindamycin) 0.039/0.117 µg/mL (*N*-demethylclindamycin) 0.042/0.126 µg/mL (clindamycin sulfoxide) 0.043/0.129 µg/mL (metronidazole) 0.045/0.135 µg/mL (hydroxymetronidazole)	[61]
*piperacillin* *tazobactam*	plasma urine	ultrafiltration filtration (0.45 µm)	UHPLC-MS/MS *m*/*z* Q1→Q3 518.0→143.0 229.0→138.0	Column: C18 Shimadzu Shim-pack XR-ODS III (50 × 2.0 mm i.d, 1.6 μm) Mobile phase: acetonitrile (0.1% FA)/water (0.1% FA) gradient elution 1 µL	0.01/0.5 µg/mL (piperacillin) 0.01/5 µg/mL (tazobactam)	[80]
*amoxicillin* *ampicillin* *piperacillin* *meropenem* *cefuroxime* *ceftazidime* *cefazolin*	human plasma	protein precipitation (ACN)	UPLC-MS/MS *m*/*z* Q1→Q3 366.16→113.94 350.16→106.00 518.26→359.09 384.18→141.03 423.09→207.00 547.22→468.10 455.16→323.00	Column: ACQUITY UPLC BEH C18 column (100 mm × 2.1 mm i.d. 1.6 μm) Mobile phase: acetonitrile (0.1% FA)/water (0.1% FA) gradient elution 0.4 mL/min, 50 °C, 10 µL	-/0.5–1.5 mg/L	[81]
*amoxicillin* *cefazolin* *cefepime* *cefotaxime* *ceftazidime* *cloxacillin* *oxacillin* *piperacillin*	human plasma	protein precipitation (ACN)	UHPLC-UV 230 nm 260 nm	Column: Hypersil Gold PFP column (100 mm × 2.1 mm i.d. 1.9 μm) Mobile phase: 10 mM phosphoric/acetonitrile gradient elution 500 µL/min, 40 °C,10 µL	-/2–100 mg/L	[82]

ACN, acetonitrile; DCM, dichloromethane; FMOC-Cl, 9-fluorenylmethyl chloroformate; LLE, liquid-liquid extraction; SDS, sodium dodecyl sulfate; SPE, solid-phase extraction; TFA, trifluoroacetic acid; SPME, solid-phase microextraction; Q1, parent ion; Q3, product ion; Ex, Excitation wavelength; Em, Emission wavelength.

**Table 3:** Identification and determination of antibiotics and their metabolites by electrophoretic method.

Antibiotic/Metabolite	Matrix	Sample Peparation	Detection	Capillary Parameters	LOD/LOQ (units)	Ref.
cefazolin cefamandol cefuroxim ceftazidim ceftriaxon cefepim	serum cerebrospinal fluid sputum	lyophilization direct injection	CZE-PD 270 nm	25 mM borate buffer (pH 9.1), 50 mM SDS L_tot_ = 48 cm, L_eff_ = 40 cm, i.d. = 50 µm 20 kV, 25 °C, 2 s	0.42–0.84/ µg/mL	[92]
sulfamethoxazole *N*^4^-acetylsulfamethoxazole trimethoprim trimethoprim 1-oxide trimethoprim 3-oxide	human serum	protein precipitation (ACN)	MEKC-DAD 260 nm 206 nm	20 mM borate buffer (pH 9.3), 25 mM SDS + 5% ACN L_tot_ = 60.2 cm, L_eff_ = 50 cm, i.d. = 75 µm 30 kV, 20 °C, 5 s	0.04–0.06/0.13–0.24 mg/L	[93]
ceftazidime cefotaxime cefuroxime ceftriaxone	wound drainage cerebrospinal fluid serum urine	filtration (0.45 µm)	CZE-UV 270 nm	25 mM borate, buffer (pH 9.2) L_tot_ = 48.5 cm, L_eff_ = 40 cm, i.d. = 50 µm 25 kV, 25 °C, 0.2 s	0.21–0.48/ µg/mL	[94]
ceftazidime	human blood	protein precipitation (ACN)	CE-DAD 200 nm 260 nm	50 mM chloroacetic acid, 20% *v*/*v* methanol, 0.5% *v*/*v* INST (pH 2.32) L_tot_ = 31.5 cm, L_eff_ = 23 cm, i.d. = 25 µm 30 kV, 25 °C, 30 s	0.42/ µg/mL	[91]
vancomycin	human serum	direct injection	MEKC-PDA 210 nm	25 mM borate buffer (pH 10.0), 100 mM SDS L_tot_ = 67 cm, L_eff_ = 50 cm, i.d. = 75 µm 25 kV, 25 °C, 4 s	1 µg/mL 1 µg/mL	[95]
daunorubicin	human plasma	SPE (MeOH)	CE-LIF 520 nm	100 mM sodium dihydrogenphosphate (pH 5.0) L_eff_ = 40 cm, i.d. = 50 µm 10 kV, 25 °C, 10 s	-/1 µg/L	[96]
cephalexi cefadroxil cefaclor ceftazidim cefsulodin cefotaxim cefamandol cefuroxim cefodizim	urine	filtration (0.2 µm)	CZE-DAD 210 nm	50 mM citrate buffer (pH 6) L_tot_ = 48.5 cm, i.d. = 50 µm 30 kV, 25 °C, 9 s	2.5–5/ µg/mL	[97]
cefadroxil cefixime cefuroxime sodium ceftriaxone sodium ceftizoxime cefaclor cefradine cefotoxime	urine	filtration (0.42 µm)	CE-UV 214 nm	50 mM sodium tetraborate buffer (pH 9.0) L_tot_ = 57 cm, L_eff_ = 50 cm, i.d. = 75 µm 30 kV, 25 °C, 4 s	0.5–5/-µg mL	[89]
moxifloxacin lomefloxacin norfloxacin ciprofloxacin ofloxacin enrofloxacin oxolinic acid flumequine	human blood	protein precipitation (MeOH)	CE-FD 240–400 nm	50 mM phosphoric acid (pH 7.55), 40% acetonitrile L_tot_ = 70 cm, L_eff_ = 55 cm, i.d. = 75 µm 50 mbar, 25 °C, 8 s	0.5–15/1.5–45 µg/L	[98]
gentamicin	smear of the wound	direct injection	CZE-DAD	TBE buffer, 0.0125% PEO (pH 8.53) L_tot_ = 33.5 cm, L_eff_ = 25 cm, i.d. = 75 µm 20 kV, 25 °C, 10 s	-/-	[99]

**Table 4:** Data of bacterial isolates from various clinical materials determination by MALDI-TOF MS.

Bacteria Speices	Clinical Samples	Matrix Solution	Sampling Technique	Identification System	Degree of Compliance Identification (%)	Ref.
*Eschericha coli* *Klebsiella pneumoniae* *Klebsiella oxytoca* *Citrobacter* spp. *Enterobacter* spp. *Pseudomonas aeruginosa* *Proteus mirabilis*	urine	HCCA	direct application	MALDI VITEK^®^ MS	86 100 67 100 75 100 100	[152]
*Eschericha coli* *Klebsiella pneumoniae* *Klebsiella oxytoca* *Enterococcus faecium* *Enterococcus faecalis* *Pseudomonas aeruginosa* *Proteus mirabilis*	urine	HCCA	protein extraction	MALDI BioTyper	95 93 100 82 90 86 98	[149]
*Staphylococcus epidermidis* *Klebsiella pneumoniae* *Eschericha coli* *Staphylococcus haemolyticus*	blood	HCCA	direct application	MALDI VITEK^®^ MS	65 97 93 80	[155]
*Lactobacillus fermentum* *Lactobacillus salivarius* *Lactobacillus rhamnosus* *Lactobacillus plantarum*	saliva	HCCA	protein extraction	MALDI BioTyper	80 36 75 100	[156]
*Staphylococcus epidermidis* *Staphylococcus hominis* *Staphylococcus haemolyticus*	blood	HCCA	protein extraction	MALDI BioTyper	99 100 100	[157]
*Mycobacterium abscessus* *Mycobacterium fortuitum* *Mycobacterium avium*	sputum pus peritoneal fluid urine	HCCA	protein extraction	MALDI BioTyper	97	[158]
*Veillonella* spp.	abdomen fluid pleural fluid bile surgical wounds pus operating material blood	HCCA	direct application	MALDI BioTyper	100	[159]
*Escherichia coli* *Streptoccocus aureus* *Staphylococcus epidermidis*	blood urine pus swab cerebrospinal fluid respiratory tract wound specimens	HCCA	protein extraction	MALDI BioTyper	100 100 100	[160]
*Aeromonas* spp.	feces	HCCA	direct application	MALDI BioTyper	100	[161]
*Streptoccocus* spp.	vitreous samples	HCCA	protein extraction	MALDI BioTyper	96	[149]
*Escherichia coli*	urine blood	HCCA	direct application protein extraction	MALDI BioTyper	94	[162]
*Eggerthella lenta*	blood	-	direct application	MALDI BioTyper MALDI VITEK^®^ MS	94 100	[163]

## Abbreviations

BGE	background electrolyte
C4D	non-contact conductivity detection
CAD	charged aerosol detector
CITP	capillary isotachophoresis
Co	cobalt
CZE	capillary zone electrophoresis
DAD	diode-array detector
ECD	electrochemical detection
ELISA	enzyme-linked immunosorbent assay
ELSD	evaporative light scattering detector
ESBL	extended-spectrum beta-lactamases
ESI	electrospray ionization
FISH	fluorescent in situ hybridization
FL	fluorescence detector
FMOC-Cl	9-fluorenylmethyl chloroformate
FPIA	fluorescence polarization immunoassay
FTIR	Fourier-transform infrared spectroscopy
GC	gas chromatography
HILIC	hydrophilic interaction liquid chromatography
HPLC	high-performance liquid chromatography
LC	liquid chromatography
LIF	laser-induced fluorescence
LLE	liquid-liquid extraction
LTA	lipoteichoic acid molecules
MALDI	matrix-assisted laser desorption/ionization
MBC	minimum bactericidal concentration
MECK	micellar capillary electrokinetic chromatography
MEPS	microextraction by packed sorbent
MIC	minimum inhibit concentration
MRSA	methicillin-resistant Staphylococcus aureus
MS	mass spectrometry detector
NACK	capillary non-aqueous electrophoresis
NMR	nuclear magnetic resonance
PBP	penicillin binding protein
PCR	polymerase chain reaction
PDA	photodiode array detector
PGD	potential gradient detection
qQq	triple quadrupole mass spectrometer
Rf	retention factor
SPE	solid phase extraction
TBA	Tris-boran-EDTA
TDM	therapeutic drug monitoring
TEA	trietyloamina
TiN	titanium nitride
TiO_2_	titanium dioxide
TLC	thin-layer liquid chromatography
TOF	time-of-flight
UHPLC	ultra-high performance liquid chromatography
UPLC	ultra-performance liquid chromatography
UV	ultraviolet
VAP	ventilator-associated pneumonia
VRE	vancomycin-resistant Enterococcus
WHO	World Health Organization
